# Targeting BCL2-Proteins for the Treatment of Solid Tumours

**DOI:** 10.1155/2014/943648

**Published:** 2014-08-27

**Authors:** Meike Vogler

**Affiliations:** Department of Biochemistry, University of Leicester, Henry-Wellcome Building, Lancaster Road, Leicester LE19HN, UK

## Abstract

Due to their central role in the regulation of apoptosis, the antiapoptotic BCL2-proteins are highly promising targets for the development of novel anticancer treatments. To this end, several strategies have been developed to inhibit BCL2, BCL-X_L_, BCL-w, and MCL1. While early clinical trials in haematological malignancies demonstrated exciting single-agent activity of BCL2-inhibitors, the response in solid tumours was limited, indicating that, in solid tumours, different strategies have to be developed in order to successfully treat patients with BCL2-inhibitors. In this review, the function of the different antiapoptotic BCL2-proteins and their role in solid tumours will be discussed. In addition, a comprehensive analysis of current small molecules targeting these antiapoptotic BCL2-proteins (e.g., ABT-737, ABT-263, ABT-199, TW-37, sabutoclax, obatoclax, and MIM1) will be provided including a discussion of the results of any clinical trials. This analysis will summarise the potential of BCL2-inhibitors for the treatment of solid tumours and will unravel novel approaches to utilise these inhibitors in clinical applications.

## 1. Mechanisms of Apoptosis

Evasion of cell death or apoptosis is a key hallmark of cancer [1]. Generally, cells can die by apoptosis, a form of programmed cell death, or after acute injury by necrosis and cell lysis, which initiates an inflammatory response. Apoptosis was first described as a unique process associated with typical morphological changes by Carl Vogt as early as 1842 and was named apoptosis in 1972 [2]. It is a common property of multicellular organisms and is present in virtually all cell types throughout the body. Apoptosis plays a fundamental role in physiological processes, especially in mammalian development and the immune system [3, 4]. In addition, apoptosis represents a major barrier to cancer cells that must be circumvented. Therefore, many tumours acquire resistance to apoptosis through a variety of strategies. The most commonly occurring loss of a proapoptotic regulator involves the p53 tumour suppressor gene [5]. In addition to the activation of proapoptotic factors, resistance to apoptosis is often due to upregulation of antiapoptotic factors. Thus, a number of genes that encode components of the apoptotic machinery are directly targeted by activating or inactivating genetic lesions in cancer cells.

In many tumours, deregulation of cell death underlies drug resistance and is a major reason for failure of conventional anticancer therapy. Upon activation, apoptosis unfolds in a precisely organised series of steps, resulting in characteristic cellular changes, including chromatin condensation, nuclear fragmentation, breakdown of the cytoskeleton, and cell shrinkage. Most of the morphological changes associated with apoptosis are caused by a set of proteases that are specifically activated in apoptotic cells [6]. These homologous endopeptidases belong to the large family of proteins called caspases (cysteine-dependent aspartate-specific protease). Caspases are among the most specific of proteases, recognizing at least four contiguous amino acids. Although the preferred tetrapeptide motif differs among caspases, the preferred specificity of cleavage for caspases can be described as X-Glu-X-Asp [7]. Besides their function in apoptosis, some members of the caspase family participate in the processing of proinflammatory cytokines [8]. Caspases involved in apoptosis are generally divided into two categories: the initiator caspases, which include caspase-2, caspase-8, caspase-9, and caspase-10, and the effector caspases, consisting of caspase-3, caspase-6, and caspase-7. An initiator caspase is characterized by an extended N-terminal prodomain of >90 amino acids, whereas an effector caspase contains only 20–30 residues in its prodomain [9]. In addition, only initiator caspases contain a caspase recruitment domain (CARD) or death effector domain (DED) preceding the catalytic domain. All caspases are synthesized in cells as catalytically inactive zymogens. During apoptosis, they are usually converted to the active form by proteolytic processing. The activation of an effector caspase is performed by an initiator caspase through cleavage at specific internal Asp residues that separate the large and the small subunits of the effector caspase. The initiator caspases, however, are autoactivated. Since the activation of an initiator caspase in cells inevitably triggers a cascade of downstream caspase activation, it has to be tightly regulated and it often requires the assembly of a multicomponent complex under apoptotic conditions. Once activated, effector caspases are responsible for the proteolytic cleavage of a broad spectrum of cellular targets, leading ultimately to cell death. Besides caspases, the cellular substrates include structural components, regulatory proteins, inhibitors of DNAses, and other proapoptotic proteins.

**Figure 1:**
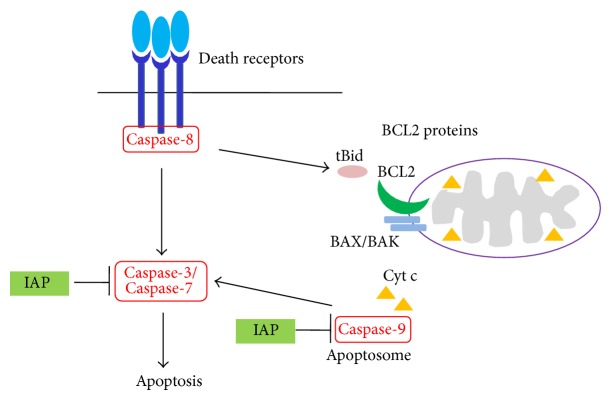
Apoptotic signalling pathways. In the extrinsic pathway, apoptosis can be initiated at the cell surface by ligation of death receptors. This results in the activation of caspase-8 at the death inducing signalling complex (DISC) and, in some circumstances, cleavage of the BH3-only protein BID. In the intrinsic pathway, apoptosis is initiated at the mitochondria and is regulated by BCL2-proteins. Activation of the intrinsic pathway, for example, by cellular stress, results in loss of mitochondrial membrane potential, release of cytochrome c, and activation of caspase-9 in the Apaf-1 containing apoptosome. Both pathways converge into the activation of the executioner caspases, for example, caspase-3. Caspases may be inhibited by the Inhibitor of apoptosis proteins (IAPs).

Apoptosis can be triggered either by activating receptors on the cell surface (the extrinsic pathway) or by the perturbation of mitochondria (the intrinsic pathway) (Figure 1).

## 2. The Extrinsic or Death Receptor Pathway of Apoptosis

In the death receptor pathway, caspase-8 is the key initiator caspase. Death receptors are members of the tumor necrosis factor (TNF) receptor superfamily and comprise a subfamily that is characterized by the intracellular death domain (DD) [10]. The most prominent death ligands are CD95-ligand/Fas-ligand, TNF*α*, and TNF-related apoptosis inducing ligand (TRAIL). Upon ligand binding, receptors oligomerize and their death domains attract the intracellular adaptor protein FADD (Fas-associated death domain protein), which, in turn, recruits the inactive proform of caspase-8 or caspase-10 via their DED. The formed multiprotein complex is called DISC (death-inducing signaling complex) [11]. The DISC contains high local concentrations of the zymogene, which leads according to the induced proximity model to autoprocessing of caspase-8. This model posits that, under crowded conditions, the low intrinsic protease activity of caspase-8 is sufficient to allow the various proenzyme molecules to mutually cleave and activate each other. In some cells, known as type I cells, the amount of active caspase-8 formed at the DISC is sufficient to initiate caspase cascade and apoptosis directly, but in type II cells, mitochondria are required to amplify the apoptotic signal [12]. Notably, the activation of caspase-8 is antagonized by FLICE-like inhibitory protein (FLIP), and caspase-9 may be inhibited by the inhibitor of apoptosis proteins (IAPs), thus providing additional levels of regulation. Both pathways result in the activation of effector caspases, proteases that are responsible for most of the morphological and biochemical changes associated with apoptosis.

## 3. The Intrinsic or Mitochondrial Pathway of Apoptosis

In the intrinsic apoptotic pathway, cytochrome *c* is released from the mitochondrial intermembrane space into the cytosol, initiating the formation of the apoptosome complex and the activation of caspase-9 as the apical caspase. Therefore, outer mitochondrial membrane (OMM) permeabilisation and release of cytochrome *c* are considered critical steps in apoptosis induction. OMM permeabilisation is regulated by members of the BCL2-protein family, which act directly, and in concert, on the OMM [13, 14].

## 4. The BCL2-Family

**Figure 2:**
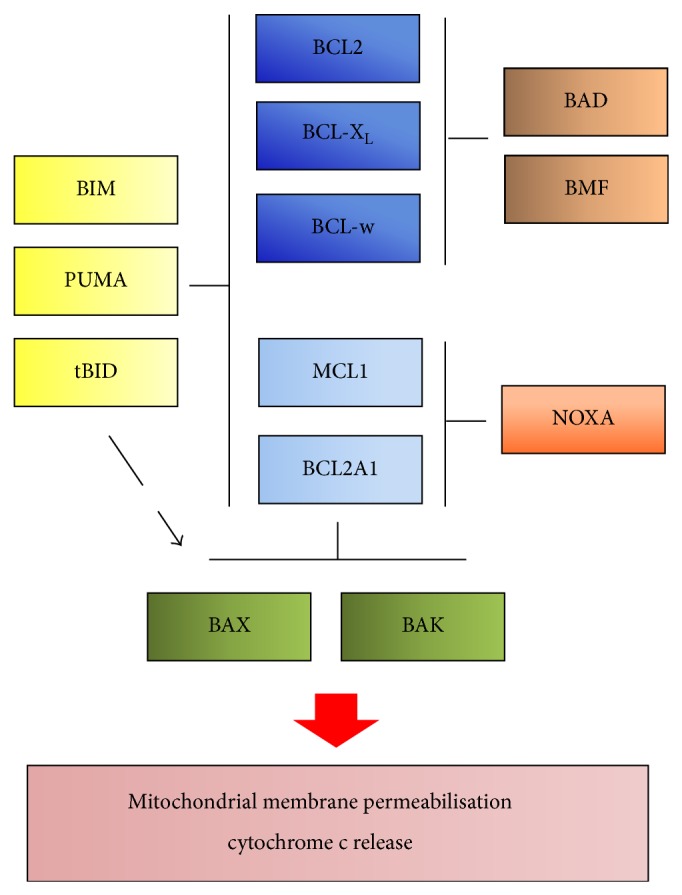
The BCL2-family. The multidomain proapoptotic proteins BAX and BAK mediate the release of cytochrome c from mitochondria into cytosol. They are inhibited by the antiapoptotic BCL2-proteins (BCL2, BCL-X_L_, BCL-w, MCL1, and BCL2A1). BH3-only proteins (e.g., BIM, BID, PUMA, BAD, BMF, and NOXA) can neutralize the function of the antiapoptotic BCL2-proteins and may also directly activate BAX and BAK.

The BCL2-family is a group of 20 proteins that are characterised by the presence of up to four sequence motifs termed BCL2 homology (BH) domains (Figure 2). The founding member, BCL2, was first identified from the *t* (14; 18) chromosomal translocation in follicular lymphoma, which brings BCL2 under the control of the immunoglobulin heavy chain promoter [15]. Later on it was identified as a mitochondria-localised protein that functions in apoptosis regulation [16]. BCL2-proteins can be divided into three groups, the antiapoptotic BCL2-proteins, the multidomain proapoptotic proteins (BAX and BAK), and proapoptotic BH3-only proteins [17]. Cells express multiple pro- and antiapoptotic BCL2-proteins, and their interactions regulate cell survival or cell death. Several models explain how the BCL2-proteins interact to regulate apoptosis. In the OMM, BAX and BAK can oligomerise and induce formation of a pore through which cytochrome *c* can be released into cytosol [18]. The antiapoptotic proteins, comprising BCL2, BCL-X_L_, MCL1, BCL-B, BCL-w, and BCL2A1, are generally integrated in the OMM. Their main function is to protect the OMM and to prevent cytochrome *c* release. The different models to explain how the BCL2-proteins interact contain some controversy as to how the antiapoptotic BCL2-proteins prevent the activation of BAX and BAK. The surface structure of the antiapoptotic BCL2-proteins contains a hydrophobic pocket into which BH3-containing proteins bind. In the direct activation model, the main function of the antiapoptotic BCL2-proteins is to sequester BH3-only proteins. Thereby, the sequestration of the BH3-only proteins BIM, BID, and perhaps PUMA is critical for apoptosis inhibition, since if released, these proteins can directly bind to BAX or BAK to initiate their activation [19]. Notably, BID is activated upon the activation of the extrinsic pathway and cleavage by caspase-8 to form tBid. The other BH3-only proteins (NOXA, BIK, BNIP1, HRK, BMF, and BAD) are called sensitizers and can displace the direct activators from the antiapoptotic BCL2-proteins, thus contributing to the initiation of apoptosis. In the indirect activation model, apart from the BH3-only proteins, also BAK and BAX can be directly sequestered and inhibited by the antiapoptotic BCL2-proteins [20–22]. Notably, due to structural differences, certain BH3-only proteins only bind to a subset of antiapoptotic BCL2-proteins, while some BH3-only proteins like BIM, PUMA, and tBid can bind all antiapoptotic BCL2-proteins. Thus, BAD and BMF bind to BCL2, BCL-X_L_, and BCL-w but not to MCL1 and BCL2A1, whereas NOXA binds to MCL1 and BCL2A1 but not to BCL2, BCL-X_L_, and BCL-w [22, 23]. This binding pattern has important therapeutic implications, since efficient apoptosis may require the simultaneous neutralisation of all antiapoptotic BCL2-proteins [24].

## 5. Expression of Antiapoptotic BCL2-Proteins in Solid Tumours

### 5.1. BCL2

BCL2 was first discovered as an oncogene in B-cell malignancies [15, 25]. However, it is also expressed in normal lymphoid cells including T-cells [26]. The importance of BCL2 for lymphoid development is highlighted by the phenotype of* Bcl2* knockout mice, which display accelerated lymphocyte apoptosis four weeks after birth [27]. Interestingly, BCL2 is also expressed in nonlymphoid tissues like the nerve system [28, 29] and epithelium [28]. A potential function of BCL2 in neuronal tumours has first been described by Reed et al. [30]. The expression of BCL2 in normal epithelium suggests that BCL2 may also be expressed in carcinoma. Evidence that BCL2 may have oncogenic potential in carcinoma was first provided in prostate cancer, where high expression of BCL2 was found in androgen-independent tumours [31]. Since then, high BCL2 expression has been reported in many different tumour types including lung cancer [32, 33], ovary cancer [34], and breast cancer [35]. The function of BCL2 in inhibiting apoptosis has been proven in many independent studies, for example, by overexpression or knockdown [36]. Paradoxically, several studies have reported that high expression of BCL2 is not associated with the level of malignancies but may even be associated with favourable prognosis, indicating that, in solid tumours, the role of BCL2 as an antiapoptotic gatekeeper is less clear than in haematological malignancies [37, 38].

### 5.2. MCL1

The second antiapoptotic BCL2-protein discovered was MCL1. It was isolated from the myeloid leukemia cell line ML-1 when undergoing differentiation and was classified as an early response gene [39]. Many reports have demonstrated that MCL1 is a highly regulated protein with a high turn-over linked to rapid proteasomal degradation [40]. Its expression can be induced by different stimuli including cytokines [41] or DNA-damage [42]. Genetic deletion of* Mcl1* results in peri-implantation embryonic lethality, suggesting that MCL1 may have a function beyond the control of apoptosis [43].

MCL1 is an important antiapoptotic oncogene that is overexpressed in the majority of cancers. In particular, multiple myeloma cells display high expression of MCL1 and appear to be dependent on MCL1 for survival [44]. Notably, gene alterations around the locus of MCL1 on 1q21 have been identified as early as 1994, when it was discovered that 1q21 is duplicated or rearranged in many types of cancer [45]. Since then, many studies have identified MCL1 as highly amplified in cancer, emphasizing its importance for carcinogenesis. With the development of next-generation sequencing, gene alterations in cancer are now investigated on a large scale with the aim of identifying driver mutations. Using next-generation sequencing, MCL1 has been identified as the most amplified gene in a screen of 3,000 individual cancers, highlighting its importance for cancer and suggesting a unique function of MCL1 amongst the antiapoptotic BCL2-proteins [46].

### 5.3. BCL-X_L_

The antiapoptotic protein BCL-X_L_ was identified only a few months after MCL1 as a BCL2-related protein isolated from chicken lymphoid cells [47]. Interestingly, the underlying gene* Bcl2l1* can be expressed in two different isoforms, encoding BCL-X_L_ and BCL-X_S_. While BCL-X_L_ has a well-described antiapoptotic function, BCL-X_S_ may be proapoptotic and its expression may counteract the antiapoptotic function of BCL2. BCL-X_L_ displays a wider tissue distribution than BCL2, and besides lymphocytes, neuronal cells and epithelium high expression is found in reproductive tissues [48]. Its importance in neuronal tissue is emphasized by the phenotype of* Bcl2l1* deficient mice, which is embryonic lethal due to massive apoptosis in the brain [49]. Elevated expression of BCL-X_L_ was found in many solid tumours including neuronal tumours [50], adenocarcinoma [51, 52], bladder cancer [53], and gastric cancer [54]. Generally, BCL-X_L_ appears to be more frequently overexpressed in solid tumours than BCL2. The importance of BCL-X_L_ in solid tumours was highlighted by a bioinformatics screen to identify markers of chemosensitivity. In this screen, mRNA expression was compared in 60 tumour cell lines and correlated with sensitivity to a panel of 122 standard chemotherapeutic drugs. The most striking relationship observed was a strong negative correlation between basal expression of BCL-X_L_ and sensitivity to drugs [55]. In addition, BCL-X_L_ was identified as one of the key genes frequently amplified in cancer [46].

### 5.4. BCL2A1

In 1993, another antiapoptotic BCL2 protein was discovered and named BCL2A1 or Bfl1 [56]. In contrast to other antiapoptotic BCL2-proteins, BCL2A1 does not display a well-defined C-terminal transmembrane domain [57] and its function in apoptosis inhibition is less well established [58]. The phenotype of* Bcl2a1* knockout mice is less severe than that of other BCL2-proteins with* Bcl2a1* deletion only resulting in hair loss during ageing [59]. However, this may be explained by the existence of multiple gene copies, and loss of BCL2A1 using* in vivo *RNAi has revealed a more severe phenotype in leukocyte development [60].

BCL2A1 is mainly expressed in lymphoid malignancies and appears to play only a minor role in solid tumours [58]. When first identified, human* BCL2A1* mRNA was found overexpressed in stomach cancer compared to normal tissue, indicating a possible function of BCL2A1 also in solid tumours [61]. Amongst a panel of different solid tumour tissues, the highest expression of* BCL2A1* mRNA was detected in breast cancer samples [62]. Interestingly, in advanced breast cancer a higher expression of* BCL2A1* mRNA was found when compared to less advanced tumours [63], suggesting an association of BCL2A1 expression with later and more severe disease stages. Furthermore,* BCL2A1* expression was associated with metastatic disease in melanoma [64] and hepatocellular carcinoma [65]. Interestingly, expression data collected in Oncomine (https://www.oncomine.org) indicate that melanoma may display higher expression of* BCL2A1* than other solid tumours, and its function in inhibiting apoptosis has recently been demonstrated using siRNA mediated knockdown, which was sufficient to induce apoptosis in the melanoma cell line 1205Lu [66]. Transcriptional profiling indicated that* BCL2A1* is highly expressed in squamous cell carcinoma of the skin [67] and that, later on,* BCL2A1 *was observed to be overexpressed in oral squamous cell carcinoma [68]. In summary, BCL2A1 has been identified as overexpressed in a variety of haematological malignancies as well as solid tumours and appears to be predominantly associated with advanced or metastatic disease stages.

### 5.5. BCL-w

BCL-w was identified as an antiapoptotic BCL2 protein in 1996 by Suzanne Cory's laboratory [69]. A study in mice lacking BCL-w indicated that, unlike other BCL2-proteins, BCL-w does not have an important function in lymphoid cells but, instead, appears to be essential for spermatogenesis [70]. However, a function of BCL-w in testicular cancer has not yet been described. Later, studies have found expression of BCL-w in the small intestine as well as epithelial tumours and established a function for BCL-w in protecting epithelial cells from apoptosis induced upon DNA-damage [71].

### 5.6. BCL-B

The last antiapoptotic BCL2-protein discovered was BCL-B in 2001 [72]. Interestingly, BCL-B binds and inhibits BAX but not BAK. Its mRNA appears to be widely expressed in human tissues. Tumour specific overexpression of BCL-B was observed in breast, gastric, colorectal, and lung adenocarcinoma and correlates with poor prognosis, indicating that BCL-B may play a prominent role in inhibiting apoptosis in solid tumours [73].

## 6. Targeting of BCL2-Protein as a Novel Anticancer Treatment

Due to their prominent role in inhibiting apoptosis, the BCL2-proteins have been recognised as promising targets for the development of novel anticancer therapeutics. The first approach to target BCL2 itself was based on an antisense RNA [74]. The resulting compound, oblimersen/genasense, was studied in clinical trials for multiple malignancies including solid tumours [75–77]. Genta, the company behind the drug, filed for FDA approval of genasense for the treatment of melanoma and chronic lymphocytic leukemia in 2003 and 2006, respectively, but the drug was rejected in both cases for lack of efficacy. The clinical failure of oblimersen may have been due to the inability of antisense molecules to efficiently inhibit protein synthesis* in vivo.*

Following up on the initial antisense method, the field subsequently advanced to use high-throughput screening of chemical libraries to identify compounds capable of binding to BCL2-proteins. Gossypol, isolated from cotton seeds and used as a male contraceptive, was recognized as binding and interacting with antiapoptotic BCL2 family members as well as inducing apoptosis [78]. The R-state of gossypol (AT-101) was licensed by Ascenta Therapeutics and tested in clinical trials. Initially described as a potent and selective inhibitor of protein kinase C [79], chelerythrine, a naturally occurring benzophenanthridine alkaloid, was subsequently identified by high-throughput screening as an inhibitor of BCL-X_L_ [80]. EM20-25, which binds to the BH3 domain of BCL2, is a derivative of HA14-1, but it lacks its effects on mitochondrial respiration [81]. However, despite the use of these inhibitors in preclinical mechanistic studies, proof for their specificity for BCL2-proteins has so far been limited [24, 82, 83]. Specific inhibitors of BCL2-proteins should induce apoptosis in a BAX/BAK-dependent manner with subsequent release of cytochrome c and activation of caspase-9. Although it has been shown that several BCL2 inhibitors might activate the intrinsic apoptotic pathway, there is little evidence that activation of this pathway is required for cell death induction.

**Figure 3:**
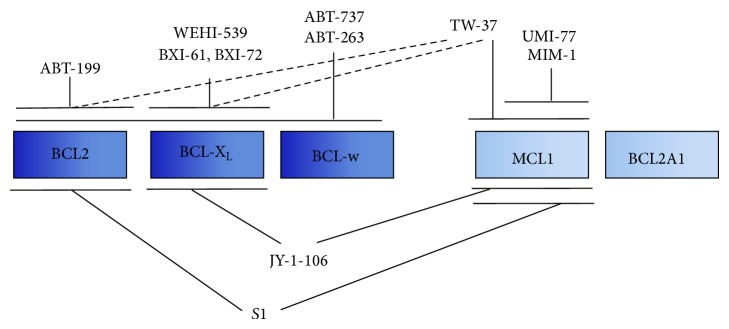
Small molecule BCL2-inhibitors. Inhibition of multiple or individual antiapoptotic BCL2-proteins by small molecule antagonists.

More recent approaches to target BCL2-protein utilise small molecule inhibitors which mimic BH3-containing proteins and bind specifically into the hydrophobic groove on the antiapoptotic BCL2-proteins (Figure 3). These small molecules are therefore also called BH3-mimetics and will be further described in the following section.

### 6.1. ABT-737

In 2005 Oltersdorf et al. published the development of ABT-737, a highly potent inhibitor of BCL2, BCL-X_L_, and BCL-w [84]. This hallmark paper, which has been cited more than 1,800 times in 2014, described how structure-activity relationship (SAR) by nuclear magnetic resonance (NMR), a technique pioneered by Shuker to identify protein ligands [85], was used to screen a chemical library for fragments that bind into the hydrophobic groove of BCL-X_L_. To achieve high-affinity binding, the proximal fragments binding to different sites in the hydrophobic groove of BCL-X_L_ have subsequently been linked, and lead compounds were developed. The final compound, ABT-737, binds with very high affinity (Ki < 1 nM) to BCL-X_L_, but due to their similar structure also to BCL2 and BCL-w. Notably, MCL1, BCL-B, and BCL2A1 have a less homologous structure and, therefore, are not inhibited by ABT-737. The potential of ABT-737 as an anticancer agent has further been demonstrated in a set of cancer cells including lung cancer cell lines. As a single agent, ABT-737 was mainly active in haematological malignancies but less active in solid tumours. A notable exception was small cell lung cancer (SCLC), where some cell lines were found to be highly sensitive to ABT-737. Additional studies have investigated the effect of ABT-737 in SCLC and identified an essential role of MCL1 in determining resistance to ABT-737 [86–88]. To this end, SCLC cell lines that have low expression of MCL1 were more sensitive to ABT-737 than those with high expression of MCL1. This relationship can be explained by the similar function of MCL1 and BCL2/BCL-X_L_/BCL-w. All of these antiapoptotic BCL2-proteins inhibit apoptosis by sequestering proapoptotic BH3-containing BCL2-proteins. In situations where the activity of BCL2/BCL-X_L_/BCL-w is inhibited due to the binding of a small molecule inhibitor (ABT-737) into their hydrophobic groove, this binding will displace any bound proapoptotic BH3-containing proteins, for example, BIM or BAX and BAK. These proapoptotic proteins are subsequently free to induce release of cytochrome c, but, in the presence of high levels of MCL1, this may now take over the function of BCL2/BCL-X_L_/BCL-w and sequester the proapoptotic BCL2-proteins previously displaced from BCL2/BCL-X_L_/BCL-w.

### 6.2. ABT-263

A major limitation of ABT-737 as an anticancer drug is that it is not orally bioavailable, which can limit the dosing regimens particularly in chronic therapy. To this end, Abbott developed a related compound, ABT-263 (Navitoclax), which is orally bioavailable and also binds to BCL2, BCL-X_L_, and BCL-w but not to MCL1, BCL2A1, and Bcl-B [89]. The biological activity of ABT-737 and ABT-263 appears to be comparable, although ABT-263 has been shown to be more readily sequestered by human serum albumin than ABT-737 [90]. ABT-263 entered clinical trials for lymphoid malignancies as well as solid tumours. While the results in haematological malignancies were encouraging and single-agent activity was reported [91], the efficacy of ABT-263 as a single agent in solid tumours was a bit disappointing. In the phase I dose-escalation study, 47 patients were enrolled, of whom 27 had SCLC [92]. In line with the preclinical data, SCLC patients appeared to respond better than the other malignancies which were not further described. In the phase II study patients with relapsed SCLC received a lead-in dose of 150 mg daily for one week followed by 325 mg of ABT-263 daily [93]. This lead-in dose was given to circumvent thrombocytopenia caused by a depletion of platelets due to their dependency on BCL-X_L_ [94]. Partial response to ABT-263 was observed in 1/39 patients (3%) while stable disease was observed in 9/37 patients (23%). Since these were chemotherapy-resistant relapsed tumour patients, the overall survival was very poor (3 months). However, the authors concluded that ABT-263 shows limited single-agent activity against advanced and recurrent SCLC [93]. Interestingly, this study also suggested a potential biomarker of clinical benefit that is based on gene amplification and copy number gain of BCL2 and colocalised genes. This highlights the potential for stratified clinical trials into which only SCLC patients with amplified BCL2 gene could be recruited.

### 6.3. ABT-199

The major toxicity of ABT-263 was an on-target effect on BCL-X_L_ expressed in platelets [94–96]. The discovery that thrombocytopenia was a major mechanism-based effect of ABT-263 led to studies that elegantly demonstrated the importance of BCL-X_L_ as a molecular clock in platelets [94]. In order to prevent this dose-limiting toxicity, Abbott reengineered ABT-263 and developed ABT-199, a small molecule inhibitor that specifically maintains binding to BCL2 but does not inhibit BCL-X_L_ or BCL-w [97]. ABT-199 efficiently induces apoptosis in BCL2-dependent tumours without causing thrombocytopenia. Impressively, a single dose of ABT-199 induced tumour lysis syndrome in three patients with leukaemia, indicating potent antitumour activity* in vivo* in humans. Therefore, ABT-199 is currently one of the most exciting agents in the clinical development for haematological malignancies. Due to the prominent role of BCL2 in B-cells, clinical trials with ABT-199 currently concentrate entirely on haematological malignancies. In solid tumours, BCL-X_L_ appears to be more important for apoptosis inhibition than BCL2, making it unlikely that a selective inhibitor of BCL2 may induce cell death. However, some tumours including SCLC display high expression of BCL2 and may be susceptible to single-agent treatment with ABT-199. Despite high expression of BCL-X_L_, ABT-199 displays preclinical activity in breast cancer cells [98]. However, a thorough analysis of the potential of ABT-199, a selective inhibitor of BCL2, or a side-by-side comparison of ABT-199 with ABT-263 or ABT-737 in solid tumours is currently lacking.

### 6.4. WEHI-539

To induce apoptosis in solid tumours, a selective inhibitor of BCL-X_L_ may be advantageous over a selective inhibitor of BCL2. Any true BCL-X_L_ inhibitor will induce platelet toxicity, but this may be manageable by applying a lead-in dosing schedule and by preselecting patients who display a low risk of developing thrombocytopenia. A specific inhibitor of BCL-X_L_, WEHI-539, was developed by experts at the Walter and Eliza Hall Institute of Medical Research (WEHI) in Australia using high-throughput screening [99]. Despite its similar function, WEHI-539 is chemically not closely related to ABT-737 and analogous compounds. It has subnanomolar binding affinity for BCL-X_L_ and induces cell death in BCL-X_L_-dependent tumour cells. To further advance in the clinical development of selective BCL-X_L_-inhibitors, Genentech and WEHI are currently collaborating to develop improved analogues of WEHI-539 [100]. These may prove particularly promising for the treatment of solid tumours, but as yet little data has been published. Support for the hypothesis that BCL-X_L_ is more relevant for solid tumours than BCL2 was provided by a recent study comparing the effect of ABT-737 (the dual BCL2/BCL-X_L_-inhibitor), ABT-199 (the selective inhibitor of BCL2), and WEHI-539 (the selective inhibitor of BCL-X_L_) in colon cancer stem cells. This study elegantly showed that ABT-737 and WEHI-539 lower the apoptotic threshold of colon cancer cells, while ABT-199 had no effect, indicating that BCL-X_L_ is the more relevant target [101].

### 6.5. BXI-61 and BXI-72

In a similar approach that was used to develop WEHI-539, another set of selective inhibitors of BCL-X_L_ were developed by screening of the National Cancer Institute (NCI) chemical library, namely, BXI-61 and BXI-72 [102]. These compounds also display subnanomolar binding affinity to BCL-X_L_ but do not bind any of the other antiapoptotic BCL2-proteins. Their efficiency as anticancer agents was investigated in lung cancer cell lines, and impressive inhibition of tumour cell growth was demonstrated* in vitro* and also* in vivo* in xenograft animal models [102]. Unfortunately, no cellular data on the specificity of the compounds was provided, and experience has shown that a lot of compounds that were originally identified as strong binders of BCL2-proteins have later on been shown to induce cell death independently of BCL2-proteins [82]. With this in mind, it is a bit worrying that, in two completely unrelated high-throughput screens, one of these compounds, BXI-61 or NSC354961, was also identified as a potential binding partner of the enzyme S-adenosylmethionine decarboxylase or an inhibitor of Hdm2 E3 ligase activity, respectively.

### 6.6. Obatoclax

In contrast to the more selective molecules described above, obatoclax or GX15-070 appears to be a pan-BCL2 inhibitor, meaning that it binds to all of the antiapoptotic BCL2-proteins [103]. Preclinical data indicate that obatoclax is not a very specific compound and induces cell death even in the absence of BAX and BAK [82]. Generally, an inhibitor that can neutralise all antiapoptotic BCL2-proteins may have therapeutic advantages, since the function of the antiapoptotic BCL2-proteins is partially redundant and efficient apoptosis may require the neutralisation of all BCL2-proteins [24]. However, such a compound is also bound to have higher toxicities. Early clinical trials with obatoclax have indicated neuronal toxicity which was not observed with more specific compounds [104]. Notably, this toxicity may depend on the dosing schedule. Due to its ability to bind MCL1, obatoclax may be particularly promising for the treatment of solid tumours. To this end, its ability as an anticancer agent was investigated in clinical trials for SCLC [105]. Results were disappointing, and obatoclax mesylate added to topotecan did not exceed the historic response rate seen with topotecan alone in patients with relapsed SCLC following the first-line platinum-based therapy. Currently, there are no open clinical trials with obatoclax in solid tumours.

### 6.7. S1

The small molecule S1 was discovered by screening of organic compound libraries for anticancer agents [106]. By binding to BCL2 and MCL1, it displaces BAK and induces apoptosis. Interestingly, S1 appears to induce cell death dependent on BAX/BAK and knockdown of BAX and BAK prevented apoptosis, indicating that S1 could be a specific inhibitor of BCL2-proteins [107]. Antitumour activity of S1 was shown in a mouse liver carcinoma xenograft model [108]. Resistance to S1 has been reported in SCLC by the activation of the MAPK/ERK pathway and the subsequent phosphorylation of BCL2 [109]. Further investigations into the biological activity of S1 would be highly desirable to determine whether this molecule could be a promising drug candidate.

### 6.8. JY-1-106

Another pan-inhibitor of antiapoptotic BCL2-proteins was published in 2013 and is called JY-1-106 [110]. This inhibitor is based on a trisacrylamide framework and reproduces the chemical nature and relative spatial projections of the key hydrophobic side chains on one face of the BH3* α*-helix. Therefore, it can inhibit both BCL-X_L_ and MCL1. In cells, it induces the displacement of BAK from both BCL-X_L_ and MCL1 followed by apoptosis. However, data on the specificity of JY-1-106 have not yet been published and its toxicity to cells lacking BAK and BAX has not been investigated. Notably, JY-1-106 was able to suppress tumour growth in a lung cancer xenograft model, indicating that it may be a promising lead compound to treat cancer.

### 6.9. Apogossypol, Apogossypolone, and Its Derivatives

Removal of the toxic aldehyde groups in the naturally occurring polyphenol, gossypol, resulted in the synthesis of apogossypol, which binds to the hydrophobic groove of BCL2 and BCL-X_L_ [78, 111]. Further substitution of the isopropyl side groups in apogossypol yielded BI97C1 (sabutoclax or ONT-701), which targets multiple antiapoptotic members, including BCL2, BCL-X_L_, MCL1, and BCL2A1. Apogossypolone is a third-generation gossypol derivative, designed to effectively target MCL1, with reduced toxicity and nonspecific reactivity. Structural derivatives of apogossypolone include BI97C10 and BI112D1 (also called BI97D6), which were designed to display a higher selectivity for MCL1 over BCL2 or BCL-X_L_ [112–114]. Both BI97C1 and BI112D1 were selective in killing cells in a BAX/BAK- and caspase-9-dependent manner [115]. However, the inhibitors lacked the potency to induce similar extents of death in BCL2-,  BCL-X_L_-, or MCL1-dependent cells, indicating that these are specific but rather weak inhibitors of BCL2-proteins. Further investigations have shown that BI97C1 and BI112D1 induce mitochondrial fragmentation rather than apoptosis [116]. In particular, BI97C1 was proposed as a promising pan-BCL2 inhibitor for further clinical development and licensed by Oncothyreon Inc. This was mainly based on a study indicating that BI97C1 may sensitise leukaemia stem cells to receptor tyrosine kinase inhibition [117]. Its potential in treating solid tumours has been shown in murine models of prostate cancer, where BI97C1 reduced tumourigenesis. Notably, in this study BI97C1 was found to block c-Met activation rather than inducing apoptosis, confirming that its main mode of action may not be via specific inhibition of BCL2-proteins and induction of apoptosis [118].

TW-37 is a second-generation benzenesulphonyl derivative of gossypol developed through computational screening and NMR that binds to MCL1 with higher affinity than to BCL2 and BCL-X_L_ [119]. Its potential as an anticancer agent has been demonstrated in prostate cancer cells and pancreatic cancer [119, 120]. In murine embryonic fibroblasts lacking BAX and BAK TW-37 did not induce cell death, whereas wild-type fibroblasts underwent apoptosis, indicating that TW-37 induces cell death in a BCL2-protein family specific manner [115]. While cellular experiments have demonstrated that TW-37 is not a very potent inhibitor of BCL2, it may have potential in targeting MCL1 since it induces cell death in MCL1-dependent lung cancer cells [115]. Therefore, TW-37 deserves further exploration to determine its potential as anticancer agent.

### 6.10. Selective MCL1-Inhibitors

Using a library of stabilised alpha-helix of BCL2 domains, the BH3 helix of MCL1 on its own was identified as a potent and exclusive MCL1 inhibitor [121]. Such stapled peptides exhibit selectivity in disrupting specific BH3-mediated interactions* in vitro*, and their sequence-dependent proapoptotic activity has been documented* in vivo* [122, 123]. The interaction of an MCL1 stapled peptide with the BH3-binding groove was employed in a competitive screen to identify MIM-1, a novel molecule that selectively targets the BH3 binding groove of MCL-1 [124]. Whereas MIM-1 exhibits BAK-dependent apoptotic activity, its potency may be limited and cell-type dependent, as it failed to induce apoptosis in two MCL1-dependent cell lines [115]. The molecule ML311 was discovered by ultrahigh-throughput screening coupled with hit optimisation [125]. This screen was designed to identify compounds that can disrupt the interaction of MCL1 with the BH3-domain of BIM. Interestingly, a secondary screen was added to identify compounds that selectively bind to MCL1 as compared to BCL-X_L_. To this end, ML311 displays 100-fold higher affinity to MCL1 than to BCL-X_L_. Some selectivity of ML311 was also indicated by the reduced activity of ML311 in BAX/BAK-deficient cells. More recently, the selective MCL1-inhibitor UMI-77 was developed by modification of the lead compound UMI-59 [126]. UMI-77 binds to the BH3-binding groove of MCL1 with Ki of 490 nmol/L and inhibits pancreatic cancer cells growth* in vitro* and* in vivo*. Specificity of UMI-77 was further demonstrated by its inability to induce cell death in cells lacking BAX and BAK expression.

Possibly the most promising lead compounds with selective binding of MCL1 have been identified by Stephen Fesik, who was leading in the chemical development of ABT-737. In contrast to other screening approaches, Fesik and colleagues used NMR-based screening of a large fragment library to identify compounds that bind to MCL1 and identified two chemically distinct hit series that bind to different sites on MCL1 [127]. Members of the two fragment classes (benzothiophene and the indole series) were merged together to produce lead compounds that bind to MCL1 with a dissociation constant of <100 nM with selectivity for MCL1 over BCL-X_L_ and BCL2. X-ray crystallography demonstrated that the hydrophobic unit of the first fragments binds in the lower part of the BH3-binding pocket of MCL1 while the core units derived from the second fragments are positioned in the upper part of the pocket. A similar fragment-based screening approach had previously led to the development of ABT-737 [84], demonstrating the potential of fragment-based drug discovery to target BCL2-proteins.

## 7. Combination of BCL2-Inhibitors with Chemotherapy

With some notable exceptions, solid tumour cells appear to be resistant to single agent treatment with BCL2-inhibitors. This lack of efficiency may partially be explained by the lack of potent pan-BCL2 inhibitors, which could simultaneously neutralise all antiapoptotic BCL2-proteins. Support for this hypothesis is provided by studies that have identified MCL1 as the major resistance factor for ABT-737 [24, 128]. Only limited data is available on selective inhibitors of MCL1, and instead many approaches have targeted the transcriptional regulation of MCL1. To this end, anthracyclines as well as inhibitors of histone deacetylases or cyclin-dependent kinases have all been shown to suppress MCL1 expression and thus sensitise ABT-737 induced apoptosis [129, 130]. However, it remains to be seen in which tumour types the simultaneous inhibition of all expressed antiapoptotic BCL2-proteins is sufficient to induce cell death and inhibit tumour growth.

Another approach to utilise BCL2-inhibitors is to combine them either with other targeted agents or with conventional chemotherapy. The rationale for the combination of BCL2-inhibitors with chemotherapy is that the toxic insults induced by chemotherapeutic agents can lead to apoptosis if the threshold to undergo apoptosis is sufficiently low [131]. Since small cell lung cancer appears to be the most susceptible to BCL2-inhibitors and some single-agent activity was observed in cell lines [84], most clinical trials with BCL2-inhibitors have concentrated on small cell lung cancer. To this end, obatoclax has been combined with carboplatin and etoposide in a phase I study in small cell lung cancer [132]. Though patient numbers were small, there was a suggestion of improved efficacy in the obatoclax-treated group. Combination of obatoclax with topotecan in a phase II trial, however, concluded that obatoclax added to topotecan did not exceed the historic response rate seen with topotecan alone in patients with relapsed small cell lung cancer following the first-line platinum-based therapy [133].

Clinical trials investigating gossypol (AT-101) in small cell lung cancer concluded that gossypol alone is not active in patients with recurrent chemosensitive disease, which may be linked to the poor potency of this inhibitor [134]. When combined with topotecan or docetaxel in relapsed or refractory small cell lung cancer, the response was limited and further enrolment of patients was not justified [135, 136]. Besides small cell lung cancer, gossypol was also investigated in prostate cancer where it was combined with docetaxel and prednisone [137]. Gossypol was well tolerated but did not extend survival. However, a potential benefit was observed in high-risk patients, indicating the potential to further explore this compound in clinical trials.

The potential of ABT-263 to sensitise to chemotherapy was demonstrated in many studies in multiple cell lines [138–140]. The combination of ABT-263 with docetaxel and gemcitabine in solid tumours has been investigated in clinical trials, but results have not been disclosed yet. The related compound, ABT-199, is currently only tested in haematological malignancies, with outstanding single-agent activity reported particularly in chronic lymphocytic leukaemia and other non-Hodgkin lymphomas. Due to the high expression of BCL-X_L_, it is not clear whether a molecule like ABT-199 that selectively targets BCL2 would be beneficial in solid tumours. The importance of BCL-X_L_ is highlighted by the observation that colon cancer stem cells can be sensitised by ABT-737 or WEHI-539, but not by ABT-199 [101]. However, overexpression and addiction to BCL2 has been reported in small cell lung cancer [33, 141] and therefore a stratified clinical trial investigating ABT-199 in patients with high BCL2-expression may be justified. Treatment of small cell lung cancer with the potent inhibitor ABT-199 may be particularly promising if combined with chemotherapy.

## 8. Combination of BCL2-Inhibitors with Other Targeted Agents

A more rational approach to utilise BCL2-inhibitors in clinical applications is the combination with other targeted agents. In contrast to standard chemotherapy which interferes with all dividing cells, these are agents specifically designed to inhibit key survival signalling pathways activated in tumour cells. The most successful targeted therapies include imatinib (a tyrosine kinase inhibitor), gefitinib (an inhibitor of epidermal growth factor receptors), sunitinib (an inhibitor of vascular endothelial growth factor), and bortezomib (a proteasome inhibitor). To this end, ABT-263 has been combined with erlotinib, a tyrosine kinase inhibitor, in a clinical trial for solid tumours. Unfortunately, results of this study have not been published yet.

Apart from the inhibition of survival signalling, BCL2-inhibitors could also be combined with other targeted agents that induce apoptosis in tumour cells. To this end, obatoclax and ABT-737 sensitize hepatoblastoma cells to death-receptor-induced apoptosis [142]. However, the potential of simultaneously targeting different arms of apoptosis signalling and combining a BCL2-inhibitor with other apoptosis-inducing agents needs to be further investigated in order to determine the potential of this approach for solid tumours.

One of the most prominent survival pathways mutated in cancer is regulated by RAF (rapidly accelerated fibrosarcoma) kinases. They participate in the RAS-RAF-MEK (Mitogen-activated protein kinase kinase)-ERK signalling cascade which is frequently hyperactivated in cancer due to mutations in BRAF or RAS. Upon hyperactivation, this pathway induces the expression of proapoptotic BH3-only proteins such as BIM, BMF, and PUMA. While inhibitors of MEK and BRAF typically induce minimal apoptosis, their predominant response is a G1 cell cycle arrest [143]. However, the BH3-only proteins induced by ERK inhibition may prime tumour cells for apoptosis, thus providing a rationale for combining an ERK or MEK inhibitor with BCL2-inhibitors. To this end, addition of ABT-737 converted the predominantly cytostatic effect of MEK inhibition to a cytotoxic effect, causing long-term tumour regression in mice xenografted with human tumour cell lines [144]. Interestingly, a synthetic lethal screen to identify genes that, when inhibited, cooperate with MEK inhibitors to effectively treat KRAS mutant tumours identified BCL-X_L_ as the most prominent target [145]. In this study, combination of the MEK inhibitor with ABT-263 led to a dramatic reduction of tumour growth in xenograft models of KRAS mutant tumours, supporting combined BCL-X_L_/MEK inhibition as a potential therapeutic approach for KRAS mutant cancers and providing a basis for stratified clinical trials. To this end, a clinical trial has recently been initiated to investigate ABT-263 in combination with the MEK-inhibitor Trametinib in KRAS mutant tumours.

Similarly, ABT-737 has been shown to sensitize melanoma cells to BRAF inhibitors only when the V600E activating mutation of BRAF is present [146]. This indicates that combination of selective BRAF inhibitors with ABT-737 or ABT-263 may increase the degree and rate of responses in previously untreated patients with V600E melanoma but not in those with acquired resistance to these agents. To follow up on these preclinical studies, a clinical trial is currently underway to investigate the potential of ABT-263 in combination with Trametinib and the BRAF-inhibitor Dabrafenib as a treatment for BRAF mutant melanoma. Taken together, these studies strongly support the investigation of BCL2-inhibitors in combination with MEK, ERK, or RAF-inhibitors, and the results of the first stratified clinical trials are eagerly awaited.

## 9. Conclusions

BCL2-proteins are one of the most prominent antiapoptotic proteins deregulated in cancer. They contribute to tumourigenesis and mediate resistance to current anticancer treatments. In recent years, several promising inhibitors of BCL2-proteins have been developed and thus the great challenge now is to explore how to best utilise these compounds in which tumour types. BCL2-inhibitors are unlikely to be as efficient as single agents, but they may be very beneficial when combined with other targeted agents. Therefore, future personalised cancer treatment will include BCL2-inhibitors in those tumours that express addiction to BCL2-proteins.
